# Methods to Enhance Verbal Communication between Individuals with Alzheimer's Disease and Their Formal and Informal Caregivers: A Systematic Review

**DOI:** 10.4061/2010/906818

**Published:** 2010-06-03

**Authors:** Mary Egan, Daniel Bérubé, Geneviève Racine, Carol Leonard, Elizabeth Rochon

**Affiliations:** ^1^School of Rehabilitation Sciences, University of Ottawa, 451 Smyth Road, Ottawa, ON, Canada K1H 8M5; ^2^School of Audiology and Speech Sciences, University of British Columbia, Vancouver, BC, Canada V6T 1Z3; ^3^Commission scolaire de la Pointe-de-l'*Î*le, Montréal, QC, Canada H1A 2T7; ^4^Department of Speech-Language Pathology, University of Toronto, ON, Canada M5G 1V7; ^5^Communication Function Laboratory, Toronto Rehabilitation Institute, Toronto, ON, Canada M5G 2A2

## Abstract

Alzheimer's disease is the leading cause of dementia in older adults. Although memory problems are the most characteristic symptom of this disorder, many individuals also experience progressive problems with communication. This systematic review investigates the effectiveness of methods to improve the verbal communication of individuals with Alzheimer's disease with their caregivers. The following databases were reviewed: PsychINFO, CINAHL, EMBASE, MEDLINE, REHABDATA, and COMDIS. The inclusion criteria were: (i) experimentally based studies, (ii) quantitative results, (iii) intervention aimed at improving verbal communication of the affected individual with a caregiver, and (iv) at least 50% of the sample having a confirmed diagnosis of Alzheimer's disease. A total of 13 studies met all of the inclusion criteria. One technique emerged as potentially effective: the use of memory aids combined with specific caregiver training programs. The strength of this evidence was restricted by methodological limitations of the studies. Both adoption of and further research on these interventions are recommended.

## 1. Introduction

Alzheimer's disease (AD) is the leading cause of dementia in older adults. Currently 4.5 million Americans have AD and this number is expected to rise to 13.2 million by the year 2050 [1]. Estimates within Canada suggest that over half a million individuals over 65 years of age have AD or a related disease [2, 3]. While memory problems are the most characteristic symptom of this disorder, many individuals also experience progressive problems with communication [4–6]. The objective of this paper was to systematically review the effectiveness of methods to improve communication between individuals with AD and their formal and informal caregivers.

The decline of communication abilities in AD is gradual and is characterized primarily by problems with object naming [7–9], coherence [10], and discourse production [11] including the use of fewer information units [12], fewer target propositions [5], and an increased proportion of pronoun use [13]. Language comprehension also worsens gradually, although phonological and syntactic skills remain preserved until the advanced stages of the disease [14, 15]. 

The deterioration of the individual's ability to communicate contributes considerably to the stress and burden of caregivers [16–18] and is often classified among the most serious stressors that caregivers must face [19, 20]. Poor communication between the caregiver and the care recipient can lead to conflicts, isolation or depression in one or both of these individuals [18, 21, 22] and may lead to earlier placement in institutions [16–18, 23]. Adoption of practices to enhance the verbal communication of individuals with AD could help mitigate these problems. Knowing which practices are effective would therefore be of interest to nursing and rehabilitative staff, as well as family members. Different methods have been proposed to maximize the potential of caregiver-patient communication and include memory aids, education and training of caregivers, and activity programs. 

Memory aids generally consist of biographical information (e.g., name, address, telephone number, etc.), photos of family members, and descriptions of important events in the life of the individual. By using images and phrases that are brief and simple, memory aids seek to capitalize on patients' automatic communication abilities, with the goal of improving the structure and quality of communication with others [24]. At the linguistic level, memory aids provide semantic support in the form of sentences, words and images, and access to other semantic information stored in long-term memory. Moreover, the provided written support can be used to compensate for certain comprehension deficits that may appear when instructions are provided verbally [25]. By offering visual cues, memory aids can also serve to remind individuals of the current task or topic of conversation, thus enabling them to better participate in the communicative interaction [26]. These cues also limit the number of choices that must be made and provide concrete topics for conversation [27]. Socially, memory aids support the *desire* to communicate, another aspect of communication that often remains intact in individuals with dementia [25].

Caregiver communication enhancement education and training techniques constitute another group of methods used to improve communication between caregivers and care recipients. An example of these methods is the program, “FOCUSED” [28]. FOCUSED is a systematic program with the goals of providing caregivers with information pertaining to AD and communication, correcting any misinformation pertaining to communicating with individuals diagnosed with this disease, and offering techniques aimed at maximizing communication potential [29]. Diverse strategies are recommended such as using close-ended or choice-based questions rather than open-ended ones, using direct and simple phrases, repeating key words and ideas in the conversation, noting a change in the topic of conversation, using direct contact (e.g., touch, eye contact), as well as utilizing comments and nonverbal cues to preserve the quality and flow of the conversation [29].

Other similar programs incorporate a variety of additional strategies including: offering positive feedback when individuals with dementia follow through on requests, giving the individuals sufficient time to answer a request or a question, and speaking with the individuals about their life and pastimes. Feedback to caregivers related to their use of specific versus general instructions, one-step instructions, and positive comments to the person with dementia is also recommended [30].

In addition, there are programs to teach caregivers tailor-made strategies to improve communication on the part of a particular person with AD. These rely on “conversation analysis” [21]. This individualized approach assists the caregiver to identify effective and noneffective conversation techniques and to use participants' strengths to improve their interactions [31]. Aspects of conversation that can be analyzed include turn-taking during a communicative interaction, topic maintenance, and resolving communication breakdowns [32]. The “Caregiver Communication Enhancement Education and Training Program” [33] uses a similar approach. 

As well, there are a number of activity-based approaches to increasing communication. These interventions may be carried out individually or in groups and use very specific (e.g., preparing a meal) or diverse activities to stimulate communication. Generally, the focus of these groups is on improving or maintaining a number of functional skills, with communication being one [34]. 

To date, there have been two systematic reviews of interventions to improve the communication of persons with AD with caregivers. In the first, no quantitative analysis of the results was included [35]. In the second, the focus was solely on strategies used by formal health care workers; the effectiveness of interventions with family and other informal care givers was not included in this review [36]. The purpose of this project was to conduct a review with the goal of identifying, based on empirical evidence, the most effective ways for enhancing the verbal communication of an individual with AD with his or her formal or informal caregiver. The outcome of interest was the verbal communication behavior of the person with AD. This outcome was chosen as the focus because it was felt to be the best measure of success in optimizing communication between these two partners.

## 2. Methodology

It was anticipated that the literature would contain only a limited number of randomized controlled trials (RCTs) investigating the efficacy of methods used to improve communication between individuals with AD and their caregivers. Therefore, it was decided that all relevant studies, regardless of the experimental method used, would be included in this review. The following inclusion criteria were adopted: (i) the study must be experimentally-based; (ii) the results must be quantitative in nature; (iii) the intervention examined by the study must be specifically designed to improve verbal communication between the individuals with dementia and their caregivers; (iv) at least 50% of the individuals sampled must have a confirmed diagnosis of AD. Where the specific dementia diagnosis of the participants was not provided, the studies were included if the majority of the participants were women, since AD is the most common dementia diagnosis among women [3]. The two exclusion criteria were that: (1) the study was published in a language other than English or French; and (2) communication outcome measures were directed exclusively at the caregiver (i.e., there was no information on the effect of the intervention on the verbal communication of the individual with dementia). 

A literature search was conducted up to July 2006 and then updated in June 2009 using the following computerized databases: PsychINFO (starting from 1872), CINAHL (starting from 1982), EMBASE (starting from 1980), MEDLINE (starting from 1966), REHABDATA, and COMDIS. Search terms were identified by a research librarian (search terms available on request). Identified articles were back-searched for additional relevant studies. As well, we consulted a database of articles relevant to communicating with individuals diagnosed with dementia that had been compiled by Kerry Byrne under the supervision of her doctoral advisor, J.B. Orange.

The review progressed as follows. First, all of the titles of articles produced by the literature search were read by two reviewers (two of the authors) who judged whether the study was likely to meet the inclusion criteria. Next, for all those judged as potential candidates by either reviewer, abstracts were obtained and read. Subsequently, the actual articles were obtained if both reviewers felt that the abstract indicated that the study was likely to meet the inclusion criteria. In cases where there was disagreement, a third individual (another author) was consulted to cast the deciding vote.

Following retrieval of the articles, the two reviewers performed a detailed analysis of each study. They determined which of the retrieved articles actually met the inclusion criteria. They then extracted the information used to summarize and evaluate each study. Advice was provided by the other authors during this process as needed. A third author calculated effect sizes (Cohen's d) and *P*-values where possible. Postintervention values of the dependent variables were used in these calculations.

Finally, based on these results, a summary recommendation was made concerning communication interventions with individuals with AD using the Strength of Recommendation Taxonomy (SORT) criteria [37]. Using these criteria, systematic reviews or high-quality RCTs provide level A evidence. According to the SORT guidelines a “high-quality RCT” is defined as including concealed allocation, blinding if possible, intention-to-treat analysis, adequate statistical power, and adequate followup (at least 80%). This allows for RCTs to be considered high quality even when patients cannot be masked to treatment allocation, a criterion that can be difficult to meet in many communication studies. Other types of studies provide level B evidence, as they may be open to threats to internal validity such as observer bias, allocation bias, placebo or Hawthorn affect, or lack of control for co-occurring treatments or maturation. Interventions supported by expert opinion only are judged as providing level C evidence.

## 3. Results

Titles and abstracts of 2000 articles were identified in the electronic searches (1296 from the original search and 704 from the updated search). Sixty-seven papers were identified as potentially appropriate to the review and were obtained and assessed. Fifty-three were excluded from the review (Table 1). Among studies with experimental methods, the most common reasons for exclusion were noninclusion of measures of communication for the affected individual during conversation with caregivers and intervention not directed at enhancing the affected individual's communication. 

 Thirteen studies reported in 14 papers met all of the inclusion criteria. Results from the Bourgeois et al. study [25] were also published in Dijkstra et al. [87]. In six of the included studies, the care recipients had an unspecified diagnosis of dementia [30]. The inclusion criteria previously described in the methodology were applied, but certain cases remained problematic. In the case of Allen-Burge et al. [88], five individuals had a diagnosis of dementia; but three had a diagnosis of “questionable dementia”. No information was given on whether “questionable” might be akin to “possible” dementia, the more standard way of stating that some, but not all, of the criteria for diagnosis are met. However, given that the MMSE scores of participants were consistent with a diagnosis of dementia, the study was included. Burgio et al. [30] posed a similar problem in that only 52.9% of the sampled experimental group had a diagnosis of dementia. Again, since the participants' MMSE scores appeared uniformly very low, we decided to include this study in the review.

Of the 13 studies included in the review, eight examined the effects of memory books, three evaluated training programs, and two reported on the outcomes of activity programs. The setting, participants, interventions, and designs of these studies are summarized in Table 2. The results of each of the included studies are displayed in Table 3. These results are discussed below by category of intervention.

### 3.1. Memory Books with Caregiver Training

Eight studies assessed the impact of memory books with varying levels of caregiver training. Of these eight studies, two were RCTs Bourgeois 2001 [25] and Burgio 2001 [30]. Neither met the SORT criteria for a high quality RCT. Of designs used in the six remaining studies, 4 were single subject multiple baseline Bourgeois 1990 [27], Bourgeois 1992 [90], Bourgeois 1996 [97], Hoerster 2001 [93], one was a pretest-posttest study Allen-Burge 2001 [88] and one was a case study Spilkin 2003 [96].

The results of one of the RCTs examining the impact of memory aids and caregiver training on participants' communication with formal caregivers indicated that, following this intervention, participants produced significantly more utterances and more informative communications during 5-minute conversations with their formal caregivers [25]. Due to the reporting methods used in the other RCT that examined memory aids and staff training Burgio 2001 [30], effect sizes for these outcomes could not be calculated. The authors noted no significant effect for group x time differences in coherent verbal interactions with formal caregivers. 

The pretest-posttest study of memory aids and staff training indicated that the percentage of time in coherent speech, percentage of time talking with others, and the number of positive statements to formal caregivers per hour all increased following intervention, but that these effects returned to pretest levels at one-month followup [88].

Two of the multiple baseline single-subject studies demonstrated improvement in on-topic statements to informal caregivers following the use of memory books and caregiver training for most of the participants [27, 90]. An additional multiple baseline single-subject study demonstrated possible improvement in on-topic statements to formal caregivers but the results were difficult to interpret due to the lack of a stable baseline [93]. The one case study of memory aids demonstrated increased maximal turns and improved topic maintenance with formal caregivers following intervention [96]. Social validation for this intervention was provided by most caregivers noting improved participant communication in all but one study [27].

Using the SORT criteria, given the above results, the use of memory aids with caregiver training would be recommended for use by clinicians with a rating strength of B. This rating is supported by results from two RCTs from which there were mixed results.

### 3.2. Education and Training

There were three studies that examined different education and training programs for the communication partner. One, a lower-quality RCT, assessed a family visit training program McCallion 1999 [94]. One pretest-posttest study assessed the impact of communication prescriptions Acton 2007 [89]. A second pretest-posttest study examined affected individuals' responses to direct or indirect listener repair responses Gentry 2007 [92].

No clinically or statistically significant changes in communication were noted in the lower-quality RCT of family visiting intervention program McCallion 1999 [94]. The pretest-posttest study of individualized communication prescriptions demonstrated a significant change in number of words per topic, but not number of total words Acton 2007 [89]. In the pretest-posttest study of indirect versus direct communication repair, all three participants demonstrated less topic changes under the indirect repair Gentry 2007 [92]. Due to the small number of participants, the significance of these findings could not be tested. 

Within these studies there was no firm support for intensive caregiver training, with the exception of individualized communication prescriptions. Overall, the strength of recommendation for this intervention was C, a result supported by one RCT.

### 3.3. Activity-Based Programming

The remaining two studies examined the effects of activity-based programming. One, a high-quality RCT, examined the effects of a walking and talking intervention Cott 2002 [91]. The other, a nonrandomized controlled trial, examined the effects of a Breakfast Club group Pietro 1998 [95]. 

In the only high-quality RCT included in this review, Cott and her colleagues [91] examined the effects of a program in which pairs of residents were encouraged to talk with one another, either while sitting or while walking. Neither condition led to better communication outcomes and both, in fact, demonstrated a tendency to worsened outcomes. Using a nonrandomized controlled trial, Pietro and Boczko [95] demonstrated the effectiveness of a daily group intervention that focused on communication stimulated by the preparation and sharing of breakfast. 

Using the SORT criteria, straight conversational or walking and talking programs would not be recommended, but a breakfast-based activity group would receive a recommendation with a rating strength of B. This rating is not supported by results from an RCT.

## 4. Discussion

Prior to discussing some generalities regarding ways of improving communicative interactions between individuals with AD and their caregivers gleaned from this, it is important first to note some important observations from our search results. First, almost one-third of the excluded papers reported the results of interventions to enhance communication between individuals with Alzheimer's disease and their communication partners but did not include a quantitative change in the verbal communication of the affected individual with a conversational partner. Rather, these studies used language measures that do not capture communicative interaction (e.g., naming), or measured aspects of caregiver communication or knowledge of recommendations. In order to convincingly demonstrate the effectiveness of interventions in improving the communication of individuals with Alzheimer's disease, future studies must include measures that reflect the communication abilities of the affected individual.

As well, among the studies that met the criterion of having a communication outcome, there were only four randomized control trials addressing this issue, and three were considered lower quality. It must be acknowledged that it is often difficult to conduct an RCT within the field of communication disorders and, as such case-studies and single-subject designs have traditionally been used. However, due to their ability to better control threats to internal validity, RCTs are regarded as the gold standard in determining the clinical efficacy of an intervention. To this end, it is suggested that future studies investigating the efficacy of recommended techniques to improve communication of persons with AD follow the RCT protocol when possible, including those criteria necessary to be considered a high-quality RCT (particularly masking of evaluators). 

In light of the above, and the fact that there were only a few studies meeting the inclusion criteria for selection, the majority of which had compromised internal validity due to methodological limitations, one must be cautious about generalizing the results of this review to clinical practice. Nonetheless, bearing in mind these methodological limitations (including weak estimates, small sample sizes, absence of control groups, nonrandom group allocation, and few instances of blinding of evaluators), trends in performances did emerge allowing us to suggest the following recommendations. 

 Memory aids demonstrated the clearest effectiveness in improving patients' discourse related to the particular topics that were linked to the memory-aids. These tools appear to be effective in enhancing topic maintenance, as evidenced in improvement in time on topic, words per topic, and fewer topic changes. This suggests that these interventions are addressing verbal attention and helping individuals focus their thoughts. 

However, memory aids did not appear to encourage generalization to other conversational topics. As well, participants' performance decreased over time where followup training was not provided. This latter finding underscores the importance of continuing training for communication partners using these tools.

Studies of combined memory aids were carried out primarily with nursing assistants and patients. Memory aids have received only limited testing with family caregivers (e.g., spouses). On the one hand, memory aids and training may be more or less effective when used by family caregivers compared to formal caregivers. Family caregivers may already be familiar with the content of memory books (e.g., family members, pets, previous occupation) and therefore able to initiate related conversation without them. On the other hand, memory books may cue the family caregiver to converse on specific topics, and these conversations may benefit from the in-depth shared knowledge of these topics. Further research on this intervention with family caregivers is recommended.

The “Family Visit Intervention Program” [94] could not be considered an effective intervention given the research available to date. There was some indication for the possible effectiveness of personalized communication prescriptions [89]. 

Only two studies of activity-based programs that included evaluation of communication outcomes were identified in our search. The Breakfast Club intervention [95] demonstrated promising results in a nonrandomized trial; a high-quality trial of this intervention is warranted. Lack of an effect of walking and talking in a high-quality RCT is likely due to the divided attention demands of the combined tasks [98]. Lack of effect of the conversation-based intervention in this study seems to indicate that increasing the opportunity to converse without other support or stimulation, such as that provided by a shared activity, may not enhance communication. 

Our results build on those of Zientz et al. [35] by demonstrating level B evidence for the effectiveness of memory aids with caregiver training. They also build on McGilton et al. [36] by extending the search beyond formal caregivers in institutional settings. 

This systematic review was carried out using recommended methods including a search strategy designed by a research librarian, and selection of studies and extraction of data by two independent reviewers. One important limitation of the review was that effect sizes could not be calculated for all outcomes due to the data presentation limitations within the original studies. Opening the inclusion criteria to allow for studies using a wide variety of experimental designs (i.e., not just RCTs) allowed us to consider the findings of a greater number of studies. However, conclusions from studies with different levels of validity may be misleading. To counteract this possibility, we indicated where the recommendations were supported by evidence from an RCT.

## 5. Conclusion

This systematic review of the efficacy of techniques used to improve communication between individuals with AD and their caregivers indicated the highest level of support for the use of memory aids combined with caregiver training. High-quality RCTs examining these interventions are still rare. Future high-quality studies of these interventions, incorporating measures of communicative interaction and measures of topic maintenance, are recommended.

## Figures and Tables

**Table 1:** Excluded studies.

Study	Reason for exclusion			
	Not a quantitative intervention study	If a quantitative study		
		No quantitative measure of change in the communication of the person with dementia	Intervention not directed at improving verbal communication of person with dementia	Less than 50% of participants have diagnosis of AD
Abrahams and Camp [38]		X		
Acton et al. 1999 [39]	X			
Alm et al. 2004 [40]		X		
Arkin, 1996 [41]			X	
Arkin, 1999 [42]		X		
Arkin, 2001 [43]			X	
Arkin and Mahendra, 2001 [44]				
Arkin et al. 2000 [45]		X		
Beach and Kramer 1999 [46]	X			
Bleathman and Morton 1992 [47]	X			
Boczko, 1994 [48]	X			
Bohling, 1991 [49]		X	X	
Bourgeois 1993 [50]			X	
Bourgeois, 2004 [51]		X	X	
Brotons and Koger 2000 [52]				
Done and Thomas, 2001[53]		X	X	X
Fried-Oken et al., 2008 [54]		X		
Friedman and Tappen, 1991 [55]		X		
Götell et al 2002 [56]				
Hendryx-Bendalov 1999 [57]	X			
Hopper 2001 [58]	X			
Hopper et al., 1998 [59]			X	
Mahendra and Arkin, 2004 [60]		X		
McPherson et al. 2001 [61]				X
Moore and Davis, 2002 [62]	X			
Murray et al., 2003 [63]			X	
Newman and Ward, 1992 [64]			X	
Normann et al., 2002 [65]	X		X	
Orange et al., 1995 [33]	X			
Orange and Colton-Hudson (1998) [21]		X		
Perkins et al., 1998 [66]	X			
Quayhagen and Quayhagen, 1989 [67]		X		
Quayhagen et al., 1995 [68]		X		
Quayhagen and Quayhagen, 1996 [69]		X		
Quayhagen et al., 2000 [70]		X		
Quayhagen and Quayhagen, 2001 [71]		X		
Ramanathan-Abbott, 1994 [72]	X			
Richter et al. 1993 [73]	X			
Richter et al. 1995 [17]	X			
Ripich et al. 1998 [29]		X		
Ripich et al. 1999 [74]		X		
Ripich, 1994 [75]		X		
Ripich et al. 1995 [28]		X		
Sabat 1991 [76]	X			
Sabat 1994 [77]	X			
Schneider and Camp, 2002 [78]		X		
Small et al., 2003 [79]	X		X	
Small et al. 1997 [80]			X	
Sobel 2001 [81]			X	
Savundranayagum et al. 2007 [82]	X			
Tappen et al. 1997 [83]	X			
Tappen et al. 2002 [84]			X	
Touzinsky 1998 [85]		X		
Watson et al. 1999 [86]	X			

**Table 2:** Included studies.

Study	Environment	Design	Sample	Intervention
Acton et al. (2007) [89]	Nursing home	Pretest posttest	9 women, 1 man diagnosed with dementia, mean age 81 years (range: 76–88); MMSE range: 2–25	Individualized communication prescriptions developed using Kitwood's strategies for enhancing social communication.

Allen-Burge et al. (2001) [88]	Nursing home	Pretest posttest	10 individuals enrolled, 8 completed;	Memory book plus training for nursing assistants (2 2-hour education session plus “hands-on” training, staff taught to monitor and record their use of communication skills.
			2 men, 6 women, mean age 76.9 (11.7) years, average MMSE 16.25 (5.39), 5 diagnosed with dementia, 3 with “questionable dementia”	

Bourgeois (1990) [27]	Community	Multiple baseline single subject	3 women, diagnosed with probable AD, aged 59–66, MMSE 12–18,	Memory book plus spouse trained to train the participant to converse using the memory book

Bourgeois (1992) [90]	Community	Multiple baseline single subject	3 women, 6 men, aged 67–93, MMSE 11–21	Memory book with or without primary caregiver trained to train the participant to converse using the memory book.

Bourgeois and Mason (1996) [24]	Daycare centre	Multiple baseline single subject	2 women, 2 men, 2 diagnosed with AD, 1 with possible AD, 1 with senile dementia, aged 74–80, MMSE 7–21	Memory wallets plus training for daycare volunteers

Bourgeois et al. (2001) [25] Dijkstra et al. (2002) [87]	Nursing home	Lower-quality RCT	Intervention group: 4 men, 29 women, mean age 85.7 (5.2), mean MMSE 11.9 (6.9)	Memory book plus training for nursing assistants (both education sessions and training during care—average of 8 sessions overall)
			Control group: 8 men, 25 women, mean age 84.3 (6.6), mean MMSE 13.1 (7.1)	

Burgio et al. (2001) [30]	Nursing home	Lower-quality	Intervention group: 9 men, 25 women, mean age 81.8 (8.9), 52.9% had a diagnosis of dementia in chart, mean MMSE 13.5 (6.7)	Memory book plus training (use of memory book plus general communication skills and hands-on training).
			Control group: 8 men, 25 women, mean age 82.4 (7.1), 72.7% had a diagnosis of dementia in chart, mean MMSE 12.9 (6.0)	

Cott et al. (2002) [91]	Nursing home	High-quality RCT	Talk only group: 10 men, 15 women, mean age 81.7 (7.34), mean MMSE 5.4 (6.0)	Talk only: conversation while sitting with another resident and an RA, 30 min/day, 5 days/week × 16 weeks
			Walk and talk group: 14 men, 16 women, mean age 83.2 (8.3), mean MMSE 6.2 (6.2)	Walk and talk: Supervised walking and conversation with another resident (prompts from RA) 30-min/day, 5 days/week × 16 weeks.
			Control group: 11 men, 8 women, mean age 79.8 (8.3), mean MMSE 6.3 (7.5)	Control group: neither intervention.

Gentry and Fisher (2007) [92]	Community-dwelling	Pretest posttest	3 men, diagnosed with Alzheimer's disease, MMSE 24, 20 and 8	Direct versus indirect listener communication repair.

Hoerster et al. (2001) [93]	Nursing home	Multiple baseline single subject	4 women aged 83–90, 2 diagnosed with AD, 1 with multi-infarct dementia, 1 with organic brain syndrome	Memory book plus investigator training of participant and instructions to caregiver.

McCallion et al., (1999) [94]	Nursing home	Lower-quality RCT	Intervention group: 2 men, 30 women, mean age 86.4 (6.6), mean MMSE 5.8 (6.3)	Family visit intervention program: 4 90-minute group education sessions plus personalized feedback to family members on their interactions with participant.
			Control group: 12 men, 22 women, mean age 85.5 (6.7), mean MMSE 8.0 (7.1)	

Pietro and Boczko (1998) [95]	Nursing home	Nonrandomized controlled trial	Intervention group: 20 participants, mean age 84.6 years (4.7), mean MMSE 15.6 (4.0) range 8–21	Breakfast club (breakfast-related activity plus structured conversation 5× /week over 12 weeks) versus conversation group.
			Control group: 20 participants, mean age 86.2 years (6.0), mean MMSE 13.8 (4.8) range 5–24	
Spilkin and Bethlehem (2003) [96]	Nursing home	Case study	1 man, 85 years of age, AD for 7 years, CDR rating of 3	Memory book, handout on basic strategies of communication given to daughter, 1-hour instructional workshop developed from conversational analysis of 2 10-minute interactions with his daughter.

**Table 3:** Summary of results.

Study	Communication outcomes
Acton et al. (2007) [89]	Number of words and number of words per topic generated during a 15-minute conversation with a nurse.
	Pre- and postapplication of the individualized prescriptions:
	Number of words: mean (SD) = 1052 (552), 1049 (492) NS
	Number of words per topic: mean (SD) = 52.7 (32.9), 78.8 (43.7) *P < .04

Allen-Burge et al. (2001) [88]	Computer assisted real-time observational data gathered for five 30-minute intervals at between 10 : 00–14 : 00 hrs and 17 : 00–19 : 00 hours over 5 days.
	Pre and post intervention and at 1-month followup:
	% of time coherent speech: 4.9, 8.4, 4.1
	% of time talking with others: 1.0, 3.7, 1.4
	Number of positive statements/hour: 1, 6, 4

Bourgeois (1990) [27]	Only 1 of the 3 spouses reported improvement in his wife's communication.

Bourgeois (1992) [90]	3 of 6 caregivers noted positive outcomes.

Bourgeois and Mason (1996) [24]	All four participants demonstrated some increase in the number of factual statements made. All 4 reduced the frequency of ambiguous statements produced. Two demonstrated a decrease in the frequency of unintelligible utterances produced and 1 demonstrated a slight decrease in perseverative utterances. One participant showed a slight increase in error utterances.

Bourgeois et al. (2001) [25] Dijkstra et al. (2002) [87]	Number and types of utterances during 5-minute conversation effect size and significance (*P < .05):
	utterances 0.60*, novel statements 0.26, ambiguous statements −0.21, questions 0.16, perseverance 0.04, errors 0.37, unintelligible 0.39, informative 2.71*, uninformative 0.19.
	words 0.26, unique words 0.31, information units 0.25, global coherence 0.22, local coherence 0.39, empty phrases −0.13, repetitions 0.31, indefinite words −0.17.

Burgio et al. (2001) [30]	No significant difference for group × time for coherent verbal interaction. Effect size not calculable.

Cott et al. (2002) [91]	Talk versus control Functional Assessment of Communication Skills for Adults subscale (FACS) effect size and significance (*P < .05):
	Talk versus control: FACS social communication −0.24; communication of basic need −0.24; overall communication −0.23
	Walk and talk versus control: FACS social communication −0.18; communication of basic need −0.21; overall communication −0.11

Gentry & Fisher (2007) [92]	All 3 participants demonstrated less topic changes when the communication partner used indirect repair than they did when the partner used direct repair. Significance not testable due to small sample (*n* = 3).

Hoester et al. (2001) [93]	2 of the 4 participants demonstrated increase in on-topic statements and decrease in off-topic statements. Effect of intervention on requests and assertions unclear due to lack of stable baseline.
	2 of 4 caregivers noted improvement in communication.

McCallion et al. (1999) [94]	Geriatric Indices of Positive Behavior (GIPB) Verbal behavior subscale effect size and significance (*P < .05): 3 months 0.22, 6 months 0.21

Pietro & Boczko (1998) [95]	Greater staff-rated Communication Outcome Measure of Functional Independence (COMFI) Scale effect size and significance (*P < .05) score change 2.36 *; final score 0.57.

Spilkin & Bethlehem (2003) [96]	Increased maximal turns, improved topic maintenance noted post intervention. Caregiver reported improved communication.
